# Practical Notes and Formulæ

**Published:** 1882-03-20

**Authors:** 


### PRACTICAL NOTES AND FORMULÆ.

**Aloes for Piles.**—Dr. Fordyce Barker advocates the use of aloes in hemorrhoids. The following formula is proposed by him:

| | | |
|---|---|---|
| ℞ | Pulv. aloes soc. | aa ℈j, |
| | Saponis castil. | |
| | Ext. hyoscyami | ʒss, |
| | Pulv. ipecac | grs. v. |

M. Ft. pil. No. xx. Sig. One morning and evening.

When the patient is anæmic he adds to the above 20 grains of the sulphate of iron. A popular and very useful aperient in piles is a combination of equal parts of the bitartrate of potassium and sulphur, given in milk. Sulphur internally exercises a most soothing influence on the inflamed tumors, more than can be fairly attributable to its aperient action. In those who have, or are predisposed to have, hemorrhoids. Dr. Barker recommends the following:

| | | |
|---|---|---|
| ℞ | Magnesiæ sulph | aa ℥ss. |
| | Magnesiæ carb | |
| | Potass. bitart. | |
| | Sulph. sublim. | |

M. Sig. From a teaspoonful to a tablespoonful of the powder in a wine-glass of sugar and water before breakfast. This powder produces a soft evacuation, without pain, even when the tumors are inflamed.—*Bulletin.*

**Styptic Colloid.**—The Chemist and Druggist (London) says that the following will instantly coagulate blood, forming a consistent clot, under which wounds will readily heal:—

| | |
|---|---|
| Collodion | 100 parts. |
| Carbolic acid | 10 parts. |
| Tannic acid | 5 parts. |
| Benzoic acid | 5 parts. |

Mix the ingredients in the above order.—*Med. and Surg. Reporter.*

**Ergotine in Pharyngitis.**—The Revue Mensuelle de Laryngologie, indicates a therapeutic method which may give good results in cases of chronic pharyngitis, complicated by exaggerated enlargement of the pharyngeal veins, and muco purulent secretions. It advises the use of—

| | | |
|---|---|---|
| ℞. | Ergotine | grs. xv. |
| | Tincture of iodine | ʒ j. |
| | Glycerine | f ʒ viiss. M. |

To be liberally applied, twice a day, on the pharynx, by means of a brush.—*Med. and Surg. Reporter.*

**Mild Tonic Bitters.**—

| | |
|---|---|
| Gentian root | 1 ounce. |
| Cardamom seed | ½ ounce. |
| Tincture of fresh orange peel | 2 to 4 drachms. |
| Alcohol | 2½ ounces. |
| Simple syrup | 2 ounces. |
| Water, sufficient to complete | 1 pint. |

Mix together the tincture, the alcohol, and five ounces of water, and with the mixture moisten the gentian and the cardamom previously reduced to a coarse powder. After twenty-four hours' contact, pack the drugs in a percolator, and exhaust them first with the alcoholic menstruum and then with enough water to complete fourteen ounces of percolate. Add to this the syrup, and filter through paper.—*Drug. Circular.*

**For Syphilis.**—Hydrargyrum and iodide of potassium for tertiary syphilis:

(A.)

℞ Hydrarg. bichlor .......... gr. j,

Potass. iodid .......... ʒij,

Tr. cardam. co ..........

Tr. gentian .......... } aa ℥ij,

M. Dose, one drachm.

(B.)

℞ Hydrarg. bichlor .......... gr. j¼,

Potass. iodid .......... ʒiij,

Tr. cardamom. co .......... ℥iij;

M. Dose, one drachm.

(C.)

℞ Hydrarg. biniod .......... gr. ss,
Potass. iodidi .......... ʒj,
Syr. sarsap. co .......... ℥ij.
M. Dose, one drachm three times a day.—*Drug. Circular.*

**Herpes Zoster.**—Dr. Boardman, in Buffalo Medical Journal, reports a case of this disease cured promptly by the following prescription. A good combination, doubtless, but too strong to use upon an extensive surface—

℞ Carbolic acid .......... ʒii,

Ol. oliv, .......... ℥i.

Signa: Rub well on the parts two or three times daily

**Kelly's Tonic**—

℞ Tr. nucis vomicæ .......... f. ʒij,

Acid. nitromuriat. dil .......... f. ʒiij,

Tr. cinch. co .......... f. ℥jss,

Tr. gent. co .......... ad f. ℥iij.

Dose, two drachms, in water, three times a day.—*Drug. Cir.*

**Macdonald on Carbolic Acid in Whooping-Cough.**—Dr. Macdonald (Edinburgh Medical Journal, 1881, p. 1094) says that on extended trial he finds carbolic acid, in doses of one-fourth of a minim to a child of six months, one-half minim for a year, and one minim for two years and upwards, to be the best remedy for whooping-cough. The whoop goes; the vomiting ceases; the paroxysms are modified in intensity and frequency. This result Dr. Macdonald believes to arise from an action similar to that of creasote on the motor fibres of the vagus of the stomach, and from a lowering of vitality of the specific germ of whooping-cough diseases. This points to the antiseptic treatment of the zymotic diseases generally.—*London Med. Record.*

**The Use of American Ash Bark.**—This agent is said to be very efficacious as an absorbent and deobstruent. It acts with special reference to the uterus. It attacks the benumbed or torpid vasc-motor nervous system, arouses it to new vitality, acts mildly but persistently in its secondary effects upon the absorbents, thus gradually reducing size, and bringing the uterus back to its normal condition. The time of cure varies from six weeks to three months. The wine should be taken in drachm doses before each meal. It yields its virtues only to a light wine, not exceeding 18 per cent. of alcohol.—*Therapeutic Gazette.*—[A good formula for using this drug in enlarged spleen and hepatic engorgement is:

℞. Wine of American ash.................. ℥iv.
Iodide potassium....................... ʒjss. M.

Teaspoonful three times per day.—ED. RECORD.

**Hamilton's Tonic.**—

℞ Strychniæ sulph ............................ gr. viij,

Cinchonidiæ sulph.......................... ℥j,

Tr. ferri chlor............................. ℥vj,

Syr. zingiberis,........................ } aa ℥xvj.

Acid. phosphoric. dil.................. }

Dose, one teaspoonful three times a day.—*Drug. Circular.*

**Anti-Rheumatic Mixture.**—(Mistura Antiarthritica.)

℞ Potassii iodidi.................................. ʒv,

Vini colchici sem............................. ℥j,

Tr. cimicifugæ rac............................ ℥ij,

Tr. stramon..................................... ℥ss,

Tr. opii camp.................................. ℥jss.

M. Dose, ʒi. three times a day.—*Drug. Circular.*

**Effervescing Mixture,**—(Dr. Draper.)

[No. 1]—℞ Acidi citrici.......................... } aa ʒiv

Ferri et quiniæ cit.................... }

Aquæ........................................ } aa f. ℥ij.

M.—Syr. limonis............................ }

[No. 2.]—℞ Potass. bicarb........................ ʒiv,

Aquæ..........................................ad ℥iv

M. One fluid drachm of each in two drachms of water, to be mixed at the time of taking.—*Drug. Circular.*

